# A cross-sectional study of *Trichinella* spp. infection in wolves (*Canis lupus*) reveals first evidence of *T. spiralis* in the species in Poland

**DOI:** 10.2478/jvetres-2025-0046

**Published:** 2025-09-17

**Authors:** Agnieszka Świątalska, Ewa Bilska-Zając, Weronika Korpysa-Dzirba, Aneta Bełcik, Michał Konrad Krzysiak, Magdalena Larska

**Affiliations:** 1 Veterinary Hygiene Laboratory, 80-316 Gdańsk, Poland; 2 Department of Parasitology and Invasive Diseases, Bee and Aquatic Animal Diseases, National Veterinary Research Institute, 24-100 Puławy, Poland; 3 Department of Parasitology and Invasive Diseases, University of Life Sciences in Lublin, 20-950 Lublin, Poland; 4 Department of Virology and Viral Animal Diseases, National Veterinary Research Institute, 24-100 Puławy, Poland

**Keywords:** wolf, reservoir, *Trichinella*, Poland

## Abstract

**Introduction:**

*Trichinella* spp. is an important zoonotic nematode parasite which infects a variety of hosts, not only including omnivorous and carnivorous animals but also herbivores. The environment and wildlife play a crucial role in nematode circulation in Poland. *Trichinella* spp. are present in prey animals, and the growth in the wolf population makes them potentially one of the major reservoirs, spreaders and/or indicators of *Trichinella* presence in their prey. The main aims of the study were to demonstrate the prevalence of *Trichinella* spp. in wolves, identify the predilection sites, and evaluate the species diversity and possible risk factors.

**Material and Methods:**

Forelimb, diaphragm and tongue muscle samples from 96 wolves from all over the country were examined by microscopy and molecular identification of parasitic isolates from them was made by multiplex PCR.

**Results:**

A total of 43 wolves (44%) were infected with *Trichinella* spp. For the first time, *T. spiralis* was detected in these animals, being noted in almost half of the cases. *Trichinella spiralis* infections were clustered in the north-west of the country.

**Conclusion:**

The high *Trichinella* prevalence in the apex predator suggests the wolf’s growing importance in the circulation and transmission of this food-borne parasite. This also indicates the importance of the disposal of carcasses to prevent the risks of animal and human exposure to this dangerous pathogen and the spread of *Trichinella* in a sylvatic environment.

## Introduction

*Trichinella* spp. are zoonotic nematodes which have been confirmed in more than 150 species of animals (15). Wild carnivores are considered the main natural reservoir, which include the Canidae, Felidae, Ursidae, Mustelidae and Procyonidae families as they are exposed to *Trichinella* infecting their prey (26, 42, 59). However, omnivorous and herbivorous animals also participate in sylvatic circulation of these parasites, and it is these which are believed to be mainly responsible for human exposure (12, 15, 41, 62, 63). The simple life cycle of this pathogen allows for frequent host changes, especially in the natural environment. The higher the number of different hosts infected with *Trichinella*, the higher the probability of carnivore infection. Wild carnivores being the largest harbour for *Trichinella*, it follows that the parasite biomass is observed to be higher in wildlife populations than in farm or domestic animals. Although, the most common human infections were caused by the consumption of pork meat containing live *Trichinella* larvae in the past, wild boar meat has taken on significance as a source of infection for human and domestic animals. With the increase of the size of the population wolves (*Canis lupus*), the largest extant member of the Canidae family, and a species native to Europe, America and Asia, their role as a possible link in the transmission of pathogens of public health concern is growing. According to the recent report of Boitani *et al*. (17), the total number of wolves in Europe (excluding Belarus and the Russian Federation) increased significantly over the five years reaching the estimated level of 21,500 animals in 2022. In Poland, wolves were exterminated until 1998, when the species became legally protected; therefore, access to samples originating from this species was limited and except for inhabitants of the Polish Carpathian Bieszczady Mountains, their abundance underrepresented the population (10, 18, 62). However, the strict protection policy in place for this carnivore, its lack of natural enemies and the availability of wild ungulate prey caused the rise of the number of these animals from 770 in 2010 to 4,328 in 2022 (72). Poland is inhabited by one of the largest wolf populations in Europe, only Romania and Italy having comparable populations (17). The European wolf population consists of nine subpopulations, of which three (Carpathian, Baltic and Central-European) cohabit Poland (17). The range of the wolf in Poland, which a few years ago was limited to the eastern part of the country, has expanded to western regions, creating a transboundary Central-European population spreading further into north-eastern Germany (17, 50). As a result, the access to grey wolf carcasses for diagnostic and epidemiological purposes is increasing, which enables researchers to obtain more information about the circulation of *Trichinella* spp. in these wild animals (43).

Grey wolves play an important role in keeping ecosystems in balance (31). Wolves hunt in packs, and the same sociality which prompts this behaviour and makes them excellent predators also makes them good facultative scavengers (43, 77). In Europe, the grey wolf diet consists mainly of medium-sized wild ungulates such as roe deer (*Capreolus capreolus*), wild boar (*Sus scrofa*) and chamois (*Rupicapra rupicapra*) but they also hunt larger animals, *e.g*. red deer (*Cervus elaphus*) and elk (*Alces alces*), and small mammals, *e.g*. beavers, rodents and hares (51, 61). Wolves occasionally may also prey on smaller carnivorous species including foxes, badgers, dogs and cats, or even demonstrate cannibalism associated with their agonistic behaviour and territorialism (43). The feeding habits of wolves predispose them to being frequently exposed to *Trichinella* infection. The presence of *Trichinella* spp. in grey wolves has been previously reported in many European countries. *Trichinella britovi* dominated in south, central and eastern European countries, *e.g*. Lithuania (31), Latvia (22), Estonia (47) and Italy (4, 35); and *T. nativa* did in northern Europe, *e.g*. Finland (1) and Russia (60). So far, *T. spiralis* has only been detected in grey wolves occasionally, *e.g*. in Croatia (8), Germany (39) and Finland; and *T. pseudospiralis* has been detected only once in these carnivores in Italy. None of the previous studies have confirmed species other than *T. britovi* in wolves in Poland. The most recent data on the infestation of these parasites in wolves date from a decade ago (10). Since then, trichinellosis in wolves in Poland has been understudied, while the population of this predatory species has at least doubled and wolves have come to be widely reported in urbanised areas in most of the country.

The aim of this work was to update the data on the prevalence of *Trichinella* spp. in grey wolves in Poland. This article is the first study reporting the presence and molecular identification of *T. spiralis* in this species in the country.

## Material and Methods

### Collection of samples

The study included skeletal muscle samples collected from 96 wolves killed in traffic accidents, found dead at the side of the carriageway as roadkill or found elsewhere in the forest in thirteen provinces of Poland. All three Polish wolf populations were sampled: the Central-European, Baltic and Carpathian (17). The collection of carcasses took place between 2023 and 2024, and was conducted in cooperation with local forest rangers, hunters from the Polish Hunting Society or local administrations. The group of wolves included 46 females and 48 males (data were missing for two individuals), aged between a few months and 9 years (data were missing on age for 27 individuals). Most of the wolves were roadkill, only a few were found dead in the forest and only one was illegally shot.

### Detection of *Trichinella* larvae

Prior to necropsy, the wolves were measured and weighed and the animal’s age was determined by its teeth. During the necropsy, the muscles of the whole forelimb, diaphragm and tongue were collected and frozen at –20°C until further analyses. The heads of 20 wolves which had previously been tested for rabies in regional laboratories were missing, and therefore the tongues were not available for examination. The samples were thawed at room temperature overnight, then 50 ± 1 g of muscle tissue was subsampled and digested using the magnetic stirrer method in accordance with the PN-EN ISO 18743:2015 Standard/A1. The samples were digested separately using artificial fluid consisting of pepsin and HCl. The digest was stirred for 30 min at 44–46°C, and subsequently *Trichinella* larvae were investigated under a microscope with 100× magnification for morphological identification. Then, the individual larvae were stored in 70% ethyl alcohol until the DNA extraction and molecular identification of species.

### Molecular species identification

Five larvae from each infected wolf were used for DNA extraction. When fewer than five larvae were isolated, all were used for DNA extraction. Larval DNA was isolated and purified using the IQtm System DNA kit (Promega, Madison, WI, USA), according to Zarlenga *et al*. (79). Purified DNA samples were stored at –20°C until their use in PCR. Multiplex PCR reactions were performed according to Zarlenga *et al*. (79) in a TProfessional thermocycler (Biometra, Analytik Jena, Jena, Germany) using five marker pairs allowing attachment of expansion segment V and internal transcribed spacer 1 and 2 gene fragments. Reaction products were separated electrophoretically in 1.5% agarose gels and stained with Simply Safe (EURx, Gdańsk, Poland). Bands of DNA in the gel were visualised under UV light. Negative controls (nuclease-free water) and positive controls (reference DNA samples from *Trichinella* Istituto Superiore di Sanità ISS3, ISS2 or ISS13 larvae) from the European Parasite Reference Laboratory were used for each PCR run.

### Data analysis

The results of *Trichinella* species identification were analysed in terms of geographical distribution of individual parasite species separately for each host species. The results obtained for particular provinces were mapped using Quantum GIS software. Since the distribution of the number of *Trichinella* larvae, body mass and body length variables were not proved to be normal by a Shapiro–Wilk test, the statistical analysis was performed using the non-parametric Kruskal–Wallis test. For other analysis which included binomial (age, *Trichinella* presence and *Trichinella* species) and categorical variables (age group, origin and body mass category), the χ^2^ and Mann–Whitney tests were used. The wolves were also categorised on the basis of their body mass by the 25^th^ and 75^th^ centiles into three groups. The division into age groups was as follows: pups (<1 year old), yearlings (1 year old), younger adults (2–3 years old) and older adults (≤4 years old). Values of P ≤ 0.05 were considered significant. The results were presented as means and 95% binomial or Poisson exact confidence interval (CI) calculated for dichotomous and countable outcomes, respectively. For statistical evaluation, STATA software version 11 (StataCorp., College Station, TX, USA) was used.

## Results

### *Trichinella* detection

**Fig. 1.**
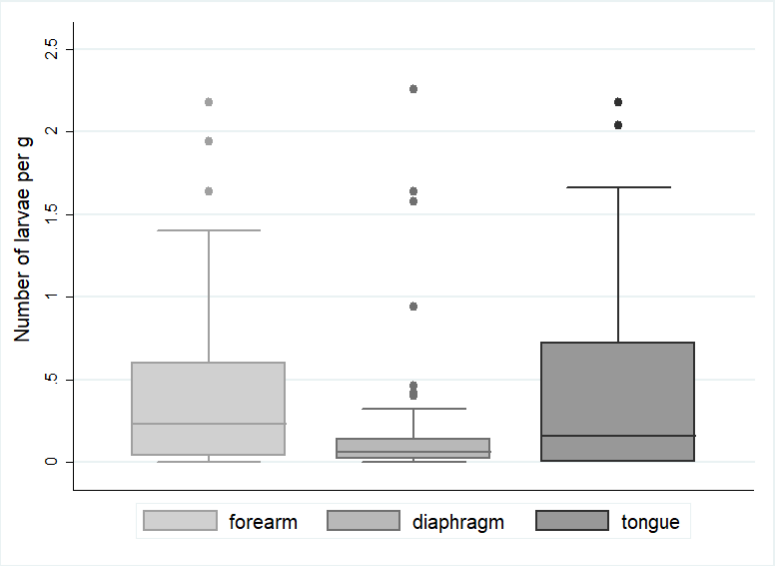
Boxplots for the number of *Trichinella* spp. larvae per gram (lpg) of three types different muscle tissues from 43 infected grey wolves. The line inside the box is the median. The top and bottom lines of the box are the 25^th^ and 75^th^ quartiles, respectively. The top and bottom whiskers mark the 5^th^ and 95^th^ quartiles, respectively. The dots represent outliers. Statistically significant differences with P-value < 0.05 are marked with asterisks

**Fig. 2.**
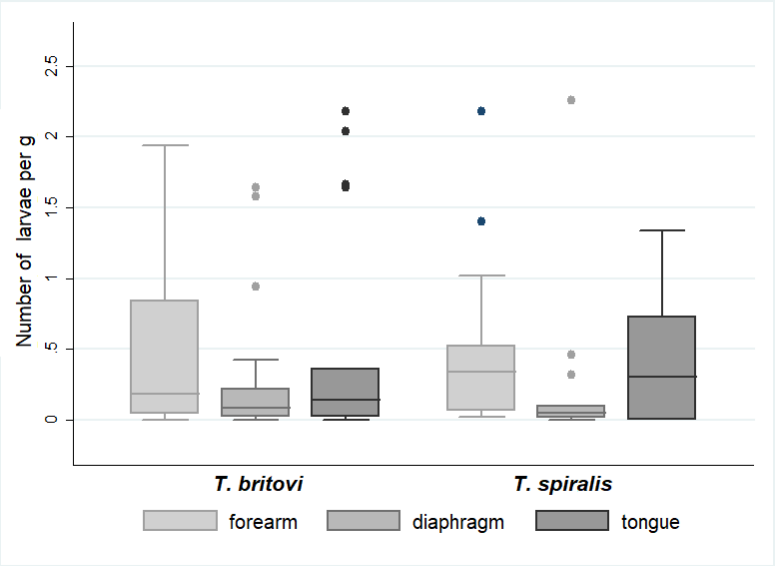
Boxplots for the number of *Trichinella* spp. larvae per gram (lpg) of three types different muscle tissues divided into *T. britovi* and *T. spiralis* from 23 and 20 infected grey wolves, respectively. The line inside the box is the median. The top and bottom lines of the box are the 25^th^ and 75^th^ quartiles, respectively. The top and bottom whiskers mark the 5^th^ and 95^th^ quartiles, respectively. The dots represent outliers. Statistically significant differences with P < 0.05 are marked with asterisks

*Trichinella* spp. larvae were detected in the muscle tissues of 43 of the 96 wolf carcasses analysed (44.8%; 95% CI: 34.6–55.3). A positive result was established on the basis of parasite larvae presence under a microscope in one of the three tested muscle tissue samples. The molecular characterisation enabled the identification of *T. britovi* and *T. spiralis* in 23 and 20 wolves, respectively. No coinfection or other species were found. Analysing the predilection site, the larvae were found in the forelimb muscles of 41 wolves (95.35%; 95% CI: 68.4–129.0), and the remaining two wolves had infection (which was with *T. britovi*) confirmed on the basis of larvae presence exclusively in tongue and diaphragm muscles. The detection rates in tongue and diaphragm muscle tissue were significantly lower: the diaphragm samples gave positive results in 34 out of 43 cases, while the tongue samples did in 25 out of 34, which resolved to sensitivities of 79.1% and 73.5%, respectively. Of the 20 wolves’ heads which were not available, 9 were from animals which tested positive; therefore, only 34 rather than 43 tongue muscle samples could be assessed. The results by species are summarised in Table 1. The overall number of larvae in the 43 wolves varied between 0.020 and 5.92 per gram of tissue (lpg), with a mean of 0.42 (95% CI: 0.31–0.55). The overall load of *Trichinella* larvae differed between the tested tissues (P-value = 0.02), with the highest mean values being in tongue tissue (0.32 lpg; 95% CI: 0.38–0.94), forelimb tissue (0.45 lpg; 95% CI: 0.27–0.71) and diaphragm tissue (0.23 lpg; 95% CI: 0.11–0.43). However, when the median value was taken into account, which is correct given the non-normal distribution of the variable, the abundance of larvae in muscle was the highest in the forelimbs (0.23 lpg), followed by the tongue (0.17 lpg), and there was a disproportionately low abundance in the diaphragm (0.06 lpg). The distribution was presented graphically in Fig. 1. Despite being apparent, statistical differences between different muscle types were only confirmed as significant between the number of larvae in forelimbs and the number in diaphragms in all cases studied (P = 0.04), and only in *T. spiralis-*harbouring muscle (P = 0.07) (Fig. 2). The differences between different muscle tissues were independent of the infecting *Trichinella* species, with the highest positivity in forelimb samples and the highest larvae burden in tongue samples (Table 1); however, these differences were statistically insignificant (Fig. 2).

**Table 1.** Sensitivities of the detection two species of *Trichinella* and the larvae burden (number larvae per gram - lpg) in three muscle tissues collected from 43 infected grey wolves

Type of muscle	*Trichinella britovi* cases			*Trichinella spiralis* cases		
	n/N (%)^a^	Mean number of larvae per g (95% CI^b^)	χ^2^ (P)^c^	n/N (%)	Mean number of larvae per g (95% CI)	χ^2^ (P)^c^
Forelimb	21/23 (91.3)	0.48 (0.24–0.86)		19/20 (95.0)	0.42 (0.18–0.83)	
Diaphragm	19/23 (82.6)	0.26 (0.1–0.57)	4.9 (0.08)	15/20 (75.0)	0.2 (0.05–0.51)	8.3 (0.02)
Tongue	13/17^d^ (76.5)	0.53 (0.24–1.0)		12/16^e^ (75.0)	0.71 (0.36–1.23)	

a – number of positive samples (n) to the number of all cases detected (N) and percentage;

b – 95% confidence interval (CI);

c – differences between the different tissue estimated by the value of chi-square tested by U Mann–Whitney test was considered significant with P-value ≤ 0.05;

d – tongue missing for six wolves;

e – tongue missing for three wolves;

### Epidemiology of *Trichinella* in wolves

**Fig. 3.**
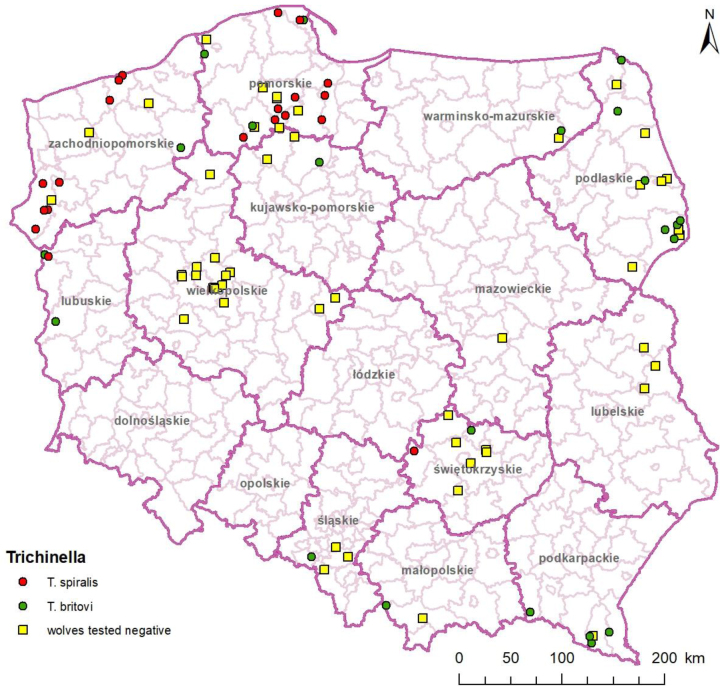
Distribution of wolves included in the study. Red dot – *T. spiralis-*positive grey wolf; green dot – *T. britovi-*positive grey wolf; yellow square – *Trichinella*-negative grey wolf

**Fig. 4.**
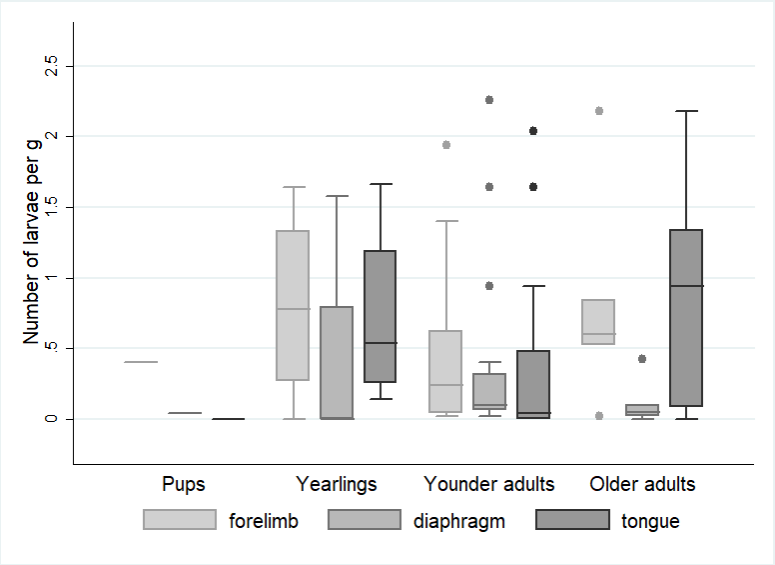
Boxplots for the number of *Trichinella* spp. larvae per gram (lpg) of three types different muscle tissues in relation to the age of wolves. The line inside the box is the median. The top and bottom lines of the box are the 25^th^ and 75^th^ quartiles, respectively. The top and bottom whiskers mark 5^th^ and 95^th^ quartiles, respectively. The dots represent data outliers. No statistically significant differences with P-value < 0.05 were found

The presence of *Trichinella* spp. larvae was confirmed in wolves in 9 out of 13 provinces (Fig. 3). Interestingly, while *T. britovi* was distributed all over the country, *T. spiralis* was detected almost exclusively only in the north-west of the country in the Zachodniopomorskie, Pomorskie and Lubuskie provinces, the outlier being a single detection in a wolf from the Świętokrzyskie province. The univariable analysis of possible risk factor for *Trichinella* infection in wolves showed the only potential significant factor was the origin (P-value = 0.002 for overall *Trichinella* and *T. spiralis* prevalence and P-value = 0.02 for *T. britovi* prevalence) (Table 2). The overall prevalence of *Trichinella* spp. in wolves was dependent on the origin region, with the highest percentages of infected wolves in the Podkarpackie (80.0%), Zachodniopomorskie (73.3%) and Lubuskie (100%) provinces (Table 2). The highest *T. britovi* prevalence was observed again in the Podkarpackie province (80%), while close to half of the wolves originating from Zachodniopomorskie and Pomorskie were infected with *T. spiralis*. There was no difference in prevalence between sexes in the overall *Trichinella* spp. prevalence and in the two species (Table 2). Although, the percentage of infected wolves increased with the age and body mass of the wolves, the differences between groups were not statistically significant. *Trichinella spiralis* larvae were found also in one year pup with 0.4 and 0.04 lpg isolated from forelimb and diaphragm muscle, respectively and negative in tongue. The prevalence in older adults (≤4 year old) and heaviest individuals (42–57 kg) reached 75% and 52.6%, respectively. Furthermore, the number of *Trichinella* larvae in different tissue did not differ significantly in different age groups (Fig. 4).

**Table 2.** Descriptive statistics of *Trichinella* prevalence among grey wolves in the study

Variable	*Trichinella* spp. prevalence			*T. britovi* prevalence			*T. spiralis* prevalence		
	n/N^a^	% (95% CI)^b^	χ^2^ (P)^c^	n/N^a^	% (95% CI)^b,d^	χ^2^ (*P*)^c^	n/N^a^	% (95% CI)^b,d^	χ^2^ (P-value)^c^
Origin (province)			30.6 (0.002)			23.9 (0.02)			31.7 (0.002)
Lubelskie	0/3	0 (0–70.8)		0/3	-		0/3	-	
Lubuskie	2/2	100 (15.8–100)		1/2	50 (1.2–98.7)		1/2	50 (1.2–98.7)	
Łódzkie	0/2	0 (0–84.2)		0/1	-		0/1	-	
Małopolskie	1/2	50.0 (12.6–98.7)		1/2	50 (1.2–98.7)		0/2	-	
Mazowieckie	0/1	0 (0–97.5)		0/1	-		0/1	-	
Podkarpackie	4/5	80.0 (28.3–99.5)		4/5	80.0 (28.3–99.5)		0/5	-	
Podlaskie	8/17	47.0 (23.0–72.2)		9/17	47.0 (23.0–72.2)		0/17	-	
Pomorskie	13/21	62.0 (38.4–81.9)		4/21	14.3 (3.0–36.3)		9/21	47.6 (25.7–70.2)	
Śląskie	1/4	25.0 (0.6–80.6)		1/4	25.0 (0.6–80.6)		0/4	-	
Świętokrzyskie	2/8	25.0 (3.2–65.1)		1/8	12.5 (0.3–52.6)		1/8	12.5 (0.3–52.6)	
Warmińsko-mazurskie	1/2	50.0 (1.2–98.7)		1/2	50 (1.2–98.7)		0/2	-	
Wielkopolskie	0/14	0 (0.0–23.2)		0/14	-		0/14	-	
Zachodnio-pomorskie	11/15	73.3 (44.9–92.2)		2/15	13.3 (4.3–48.0)		8/15	53.3 (26.6–78.7)	
Sex			1.9 (0.2)			0.01 (0.9)			0.2 (0.7)
Female	20/46	43.5 (29.9–58.9)		11/46	23.9 (12.6–38.8)		9/46	19.6 (9.3–33.9)	
Male	23/48	47.9 (33.3–62.8)		12/48	25.0 (13.6–39.6)		11/48	22.9 (12.0–37.3)	
Age group			4.5 (0.2)			1.8 (0.6)			4.7 (0.2)
Pups	1/5	20.0 (0.5–71.6)		0/5	0 (0.0-52.2)		1/5	20.0 (0.5-71.6)	
Yearlings	4/10	40.0 (12.1–73.8)		3/10	30.0 (6.7–65.2)		1/10	10.0 (0.2–44.5)	
Younger adults	19/46	41.3 (27.0–56.8)		10/46	21.7 (10.9–36.4)		9/46	19.6 (9.3–33.9)	
Older adults	6/8	75.0 (34.9–96.8)		2/8	25.0 (3.2–65.1)		4/8	50.0 (15.7–84.3)	
Body mass class			1.9 (0.4)			0.9 (0.8)			1.6 (0.4)
Lower (14–25 kg)	7/22	31.8 (13.8–54.9)		5/22	22.7 (7.8–45.4)		2/22	9.1 (1.1–29.2)	
Medium (26–41 kg)	19/43	44.2 (29.1–60.1)		10/43	23.2 (11.8–38.6)		9/43	20.9 (10.0–36.0)	
Higher (42–57 kg)	10/19	52.6 (28.9–75.5)		6/19	31.6 (12.6–56.6)		4/19	21.0 (6.0–45.6)	

a – number of positive samples/all samples tested;

b – Clopper–Pearson 95% confidence interval (CI) for binomial distribution (one-sided 97.5% for the mean = 0 and 100);

c – value of chi-square test was considered significant with P ≤ 0.05;

d – 0% values were omitted;

## Discussion

The high level of forestation, the abundance of game which is the basic food supply for wolves, the lack of natural enemies in the wild and the complete protection of the species have created ideal conditions for the expansion of the wolf population in Poland. As an excellent predator but also a scavenger, every wolf is likely to be exposed to feed that may contain *Trichinella* larvae during its lifetime. The prevalence of these parasites in wild carnivores therefore depends on the population size of and *Trichinella* species prevalence in infected animals from other host groups in a given area. Wolves are not only considered an important reservoir and carrier of *Trichinella*, but also an indicator species (74) for parasite environmental contamination and human risk exposure (44). The overall prevalence of *Trichinella* spp. of 44.8% in the tested wolves in this study was comparable with those in previous reports in the country (Table 3) (10, 18, 51). However, while the studies revealed *Trichinella* infection in more than half of wolves, they only sought to identify *T. britovi* larvae in a rather limited group of animals in terms of numbers and origin (10, 18, 26, 51). Similar infection rates were recorded in Serbia (46.5–49.5%), Bosnia and Herzegovina (38.89%) and Finland (33–39.2%) (Table 3). Interestingly, in the countries where the wolf population size is as large as in Poland, such as Italy, Romania or neighbouring Germany, the *Trichinella* prevalence in the species is in general lower, especially in the studies that used a representative number of animals. The Italian prevalence ranged from 8.9% to 31.0% (3, 4, 24, 43, 61, 69, 71), the Romanian from 30.5% to 31.0% (16, 25) and the German from 3.70% to 4.04% (30, 39). Notably higher prevalence was recorded in wolves in Latvia, where it was 69%–100% (6, 7, 22); in Estonia, where researchers noted 63.2–79.4% (29, 54); and in Russia, where up to 97.5% of wolves carried *Trichinella* (20, 54, 60) (Table 3). In some other part of Europe, Spain being an example, the incidence of *Trichinella* in wolves was low; however, these reports are quite dated and based on limited numbers of wolves (Table 3). A fairly recent report from Sweden, where the wolf population is quite small and still regulated by culling (36), showed a very low prevalence of *Trichinella* (5.6%) (40) (Table 3). In our study, the prevalence differed significantly between different regions of Poland, which may be connected to the different prey structure, biology and parasite circulation in the relevant local environments. Attention should also be paid to climatic variables, as increasing temperatures and reduction of environmental humidity may lower the survival time of larvae in host carcasses (58). On the other hand, environmental changes may influence the biology and ecology of the main host species, reducing their number and changing their age composition, the changes having the possible forms of habitat fragmentation due to the expansion of the wildlife–human interface or shortening of trophic chains, inducing stress, which may lead to host–parasite disequilibrium. Parasitic invasions are flagship examples of climate-sensitive infections, which can trigger outbreaks or epidemics caused by opportunistic and neglected parasites (34).

**Table 3.** Summary of *Trichinella* spp. prevalence reports in wolves in North America, Asia and Europe by country, period, parasite species assigned and sample type

Country (region)	Source (Reference)	Sampling period	Sample size	*Trichinella* spp. prevalence (%)	*Trichinella* species^a^	Tissue^b^
Finland	(1)	1995–2005	102	39.2	Tn (Tb, Ts)	m/d/fl
Finland	(53)	2011–2013	85	34.1	Tn (Tb, Ts)	no data
Finland	(52)	1996–1998	18	33.0	Tn, Tb	no data
Sweden	(40)	2014–2019	197	5.6	Tn, Tb	no data
Latvia	(22)	2010–2014	23	100.0	Tb	fl
Latvia	(6)	2003–2008	no data	69.0	no data	no data
Latvia	(7)	2010–2012	8	69.7	no data	m
Estonia	(29)	1992–1999	34	79.4	Tn, Tb	m
Romania (Carpathians)	(16)	2000–2005	35	31.0	Tb	m
Romania (Central)	(25)	1991	399	30.5	Tb	no data
Romania (Transylvania)	(25)	1999–2002	7	71.4	Tb	no data
Romania (Transylvania)	(25)	2014–2015	3	66.7	Tb	no data
USA and Canada	(37)	2001–2013	244	51.0	T6, Tn	tg
Canada (North) and USA	(54)	2022	no data	13–52	T6, Tn	no data
Greenland	(54)	no data	no data	50.0	T6, Tn	no data
Estonia	(54)	1992–1996	no data	75–79	T6, Tn	no data
Estonia	(54)	1999	no data	63.20	T6, Tn	no data
Finland, Norway, Russia (Kola Peninsula), Sweden	(54)	no data	no data	32–39	T6, Tn	no data
Russia	(54)	no data	no data	10–65	T6, Tn	no data
Russia	(60)	1998–2000	82	97.50	Tn, Tb	fl
Russia	(20)	1998–2000	75	97.30	Tn, Tb^c^	fl
Serbia	(81)	2009–2010	116	46.50	Tb, Ts	m
Serbia, Bosnia and Herzegovina, Macedonia	(74)	2006–2013	116	46.5	Tb	tg
Serbia	(74)	2011–2022		49.50	Tb	no data
Bosnia and Herzegovina	(55)	2013–2023	36	38.89	Tb	m
Canada (Alberta)	(27)	1975–1977	217	5.50	no data	tg, m, d
Canada (North)	(38)	2001–2015	81	62.00	no data	m, tg
Kazakhstan	(75)	2020–2023	83	20.50	Tn	m, d
Kazakhstan	(2)	2013–2023	98	20.40	no data	m
Poland	(51)	1999–2007	10	40.0	Tb	no data
Poland	(10)	1999–2015	21	54.50	Tb	m, d, fl
Poland (Carpathians)	(18)	1996–2004	6	50.00	Tb	no data
Italy	(24)	2008–2012	67	8.9	Tb	m
Italy	(4)	2004–2014	218	27.1	Tb	m
Italy	(71)	1987–1993	48	19.0	Tb	d
Italy	(69)	1991–1993	25	28.0	Tb	d, fl
Italy	(61)	1985–1995	81	31.0	Tb	m
Italy	(3)	2015–2020	213	28.0	Tb	d, fl
Italy (Western Alps)	(43)	2017–2022	130	11.53	Tb	d
Slovakia (Tatras)	(28)	2005–2006	4	0	no data	d, fl
Spain	(67)	1996–1999	47	12.8	Tb	m
Croatia	(8)	1996–2007	67	31.0	Tb, Ts	m
Germany	(39)	2007–2014	53	3.70	Tb, Ts	d
Germany	(30)	2013–2023	545	4.04	Tb, Ts	d

a – Tn: *Trichinella nativa*, Ts: *T. spiralis*, Tb: *T. britovi;*

b – d: diaphragm, fl: forelimbs, m: undetermined muscle;

c – in one individual;

The overall number of *Trichinella* larvae in the muscle tissue of 43 wolves varied between 0.020–5.92 per gram of tissue, with a mean of 0.42, which suggested less extensive invasion than was found in previous studies, among others in those of Bień *et al*. (0.009–27 lpg) in Poland (10), Omeragić *et al*. (mean 6.9, range 0.6–33 lpg) in Bosnia and Herzegovina (55) and Martinez-Carrasco *et al*. (range 0.8–45 lpg) (43), Scancerlli *et al*. (range 1–1,070 lpg) (68) and Ricchiutti *et al*. (mean 4.5 lpg) in Italy (65). However, each of these studies reported *T. britovi* or *T. pseudospiralis* infections but not any of *T. spiralis*. Pozio (61) pointed out that the extent of infection with *T. britovi* was higher than that of infection with *T. spiralis* and explained that by the different characteristics of these nematode species (57), which was confirmed in the studies of Omeragic *et al*. (55) and Blaga *et al*. (16). The findings of our study do not fit this hypothesis, because the mean lpg for *T. spiralis*- infected wolves was slightly higher than that for *T. britovi* infection (although the difference was not statistically significant). We found, however, statistical significance in the difference between the larval burdens in three different muscles of wolves infected with any *Trichinella* species, higher potential to be the predilection site for *Trichinella* larvae in wolves emerging for the forelimb and tongue muscles. However, the frequency of parasite detection in the latter might have been unrepresentatively low because of the impossibility of testing the some animal heads because they had already been removed for rabies testing and because of some technical difficulties in sample processing to those encountered by Sharma *et al*. (70). Our results correspond to findings by Ricchiutti *et al*. (65), who reported a higher larval burden in tibial muscle, with a mean of 36.9 lpg (39–1,070), than in diaphragm pillars, in which the mean burden was 8.7 lpg (1–174). Also Sharma *et al*. (70) found that wolverine (*Gulo gulo*) diaphragm samples showed much lower larvae burdens than tongue samples. Similarly to them, we also did not find any correlation of the age or sex of the animal with the *Trichinella* prevalence or larvae load, except for in the pups. This is also confirmed in the investigation among Alaskan wolves by Zarnke *et al*. (80). Several researchers have tried to determine the relevance of the differences in location of larvae, and Kapel *et al*. (32) concluded that it may be dose-dependently related to the way a species obtains food from another species which is a *Trichinella* host, which implies a lifestyle factor or the actual involvement of particular muscles as an animal acquires and consumes food. Kozar and Kozar (33) tried to confirm the greater affinity of the *Trichinella* larvae for the muscle parts which work more intensely by initiating increased exercise in mice. The results obtained have important diagnostic significance, indicating that the appropriate muscle should be selected for the detection of *Trichinella* larvae. Despite the detection of the highest number of larvae in the tongue, it seems that a better choice for the researcher should be the forelimb muscles because of the consistently higher lpg level and lack of problems with its digestion; they offer greater ease of preparation than the wolf tongue observed in the study which was not digested easily.

This is the first report on the occurrence of *T. spiralis* in wolves in Poland, and one of few in Europe (Table 3). The infection of almost half (45%) of Polish wolves with *T. spiralis* suggested changes occurring in the wolf-trichinella-environment triad. Such a high *T. spiralis* prevalence in wolves had never been reported before, and this is the first evidence of this parasite species in wolves in Poland (10, 18). Ricchiuti *et al*. (65) summarised extant studies and found that in a total of 488 wolves from European countries, 67.8% were identified as infected with *T. britovi*, while only 2.5% were found to be infected with *T. spiralis*. These latter originated from Croatia, Finland, Germany, Serbia and Spain (Table 3). In these countries (except Finland), *T. spiralis* is constantly circulating domestically or has only been eradicated quite recently, and more importantly, it is also actually detected in the sylvatic environment in a higher percentage of wild boars than *T. britovi* is (5, 23, 30, 76). In Poland, *Trichinella* has been almost eradicated from pigs, leaving the prevalence at 0.000088% (14). It is, however, still at 0.3% prevalence in wild boars, with the predominant species being *T. spiralis* (13). The high burden of the larvae in wild boar biomass and high number and density of the wild boar population in Poland factor into the maintenance of the circulation of *T. spiralis* in the environment. In the study, the high prevalence of *T. spiralis* in wolves discovered in north-western Poland coincided with the endemic occurrence of this parasite species in wild boar in the area and adjacent areas of Germany (11, 30). The prevalence of *T. spiralis* in the wild boar population in this region certainly has an impact on the spread of this nematode species in wildlife, as evidenced by the detection of *T. spiralis* in the foxes from this area (13). The occurrence of *T. britovi* in wolves in north-eastern (Podlaskie) and south-eastern (Podkarpackie) provinces is also consistent with the higher ratio of *T. britovi* to *T. spiralis* in wild boar and foxes in these provinces in relation to the north-western part of the country (13). Therefore, not only the prevalence but also the nematode species identified in wolves will depend on which host species are available prey and on the prevalence and larvae load in that prey.

The significance of wolves in *Trichinella* transmission is not fully understood. As an apex predator often preying on wild boar, they should be considered as a good indicator of the *Trichinella* presence in the environment, especially in Poland, where the nematode’s circulation is now concentrated mostly in the sylvatic cycle. However, while wild boar, red foxes and rodents are considered the main reservoirs responsible for the maintenance of *Trichinella* spp. in the environment, wolves may contribute to its long-distance dispersal as they may migrate hundreds of kilometres to establish new packs or populations or in search of an adequate food base (48, 56, 73). Also because of their growing numbers in the country, wolves are increasingly approaching human settlements, which may be an increasing public health concern (44, 65). The wolves included in the study mostly died in traffic accidents; therefore, they may mainly represent migratory individuals in dispersal, often referred to as compromised. Wolves generally live and prey in packs, and their survival is dependent on their social skills and certain behaviour such as evasion of humans. Therefore, we should consider that the wolves studied did not accurately represent the entire population, and that the noted prevalence of *Trichinella* may correspondingly be unrepresentative, because such individuals usually did not hunt on their own when they had a pack and now they are outside it, more often feed on smaller mammals or carrion. They are observed preying and scavenging on wild boar, which, in our opinion, may be the explanation for the high percentage of these wolves infected with *T. spiralis*. While wolf exposure to *Trichinella* is associated with the prevalence in their prey, there may be a possibility of spill-back from the predator to the parasite maintenance host, albeit probably more incidental than regular in occurrence (43). No data is available on wild boar scavenging on wolves, and some reports suggested that wild boar would not feed on wolves because of their fear of the large predator, contrasting this with boars’ ready scavenging on foxes (64). However, a wolf carcass may undoubtedly be consumed by other scavengers such as ravens, small rodents or other predators like foxes, badgers, raccoon dogs, bears and even other wolves, from which a risk derives for further *Trichinella* transmission and new outbreaks of trichinellosis. In the situation when almost 45% of wolves are infected by either *T. britovi* or *T. spiralis*, such transmission is quite likely to occur. Another possible reservoir of *T. spiralis* and other species, albeit one not yet reported, are beavers (66), which increasingly fall prey to wolves and constitute as much as 45% of their diet in some areas of the country (51). The last question is the impact of *Trichinella* infection on wolves’ health. Wolves are still protected species under the Bern Convention, despite their status having been downgraded from “strictly protected” to “protected” quite recently. Unlike humans, for whom trichinellosis has serious clinical consequences, animals rarely show any clinical signs of the disease (59). The parasite may be associated with abnormal behaviour because of the pain or discomfort caused by larvae migrating trough the muscle tissue, as was suspected for aggressive grizzly bears (78). Such impact was also suggested of exposure to another parasite, *Toxoplasma gondii*, which altered the behaviour of wolves so that they took more risks, including dispersing from their pack or seeking to become pack leaders (45). Hence, the ecological effect of *Trichinella* infection in wolves needs further studies.

## Conclusion

Since the carcasses of wolves and other carnivores may be a source of *Trichinella* larvae which are relatively resistant to environmental conditions (57, 66), a recommendation to remove and dispose of them should be made as a prevention measure to limit the pathogen circulation and the further exposure of humans and animals to infection.
